# ASPICov: An automated pipeline for identification of SARS-Cov2 nucleotidic variants

**DOI:** 10.1371/journal.pone.0262953

**Published:** 2022-01-26

**Authors:** Valentin Tilloy, Pierre Cuzin, Laura Leroi, Emilie Guérin, Patrick Durand, Sophie Alain

**Affiliations:** 1 Centre National de Référence des Herpèsvirus, CHU Dupuytren, Limoges, France; 2 UF9481 Bioinformatique, CHU Dupuytren, Limoges, France; 3 UF8843 Génomique médicale, CHU Dupuytren, Limoges, France; 4 IFREMER-IRSI-Service de Bioinformatique, Centre Bretagne, Plouzane, France; University of Salemo, ITALY

## Abstract

ASPICov was developed to provide a rapid, reliable and complete analysis of NGS SARS-Cov2 samples to the biologist. This broad application tool allows to process samples from either capture or amplicon strategy and Illumina or Ion Torrent technology. To ensure FAIR data analysis, this Nextflow pipeline follows nf-core guidelines and use Singularity containers. Pipeline is implemented and available at https://gitlab.com/vtilloy/aspicov.

## Introduction

Whole-genome sequencing (WGS) is used for clinical surveillance of SARS-Cov2 in order to detect emerging variants especially variants of interest (VOI) or variants of concern (VOC), to facilitate epidemiological studies and to anticipate possible therapeutic/vaccinal escape.

Two main library sequencing preparation methods are used according to the context and sample origin: shotgun metagenomics and target enrichment. Various ways are undertaken such as transcriptome sequencing or combination of strategies (hybrid capture enrichment, …) depending on goals and context [1,2].

Shotgun metagenomics method is used to capture SARS-Cov2 sequences by hybridization from a highly concentrated sample. Target enrichment or amplicon strategy is often chosen to amplify and detect SARS-Cov2 at low concentrations such as in wastewaters and some particular samples (stools, blood, end-infection steps samples, …). It is also important to consider NGS sequencing platform which will not provide the same sets of data and/or which are optimized for a particular strategy library kit.

In order to cover a large range of sequencing technologies and handle all parameters of our analysis we developed ASPICov, a pipeline able to identify whole genome variations at the nucleotide or amino-acid level in samples using a reference sequence. This pipeline is a multistep Nextflow [3] pipeline able to process raw-reads sequences into usable information such as quality reports, VCF files, sequence consensus and plots (variants and coverage).

## Material and methods

### Implementation

ASPICov workflow was created as a Nextflow pipeline following some of the nf-core standards requirements to setup a portable pipeline. Code wrapping the many tools used in ASPICov (see below) is written in bash and Python. Tools themselves have been integrated into ready-to-use Singularity containers [4]. Singularity definition files (used to build images) as well as binary images are all available for download (see below). ASPICov comes with a test data set. In such a way, users can validate the correct execution of ASPICov on their computing infrastructure after cloning the pipeline from its public Gitlab repository.

### Pipeline steps and tools used

**Fig 1:**
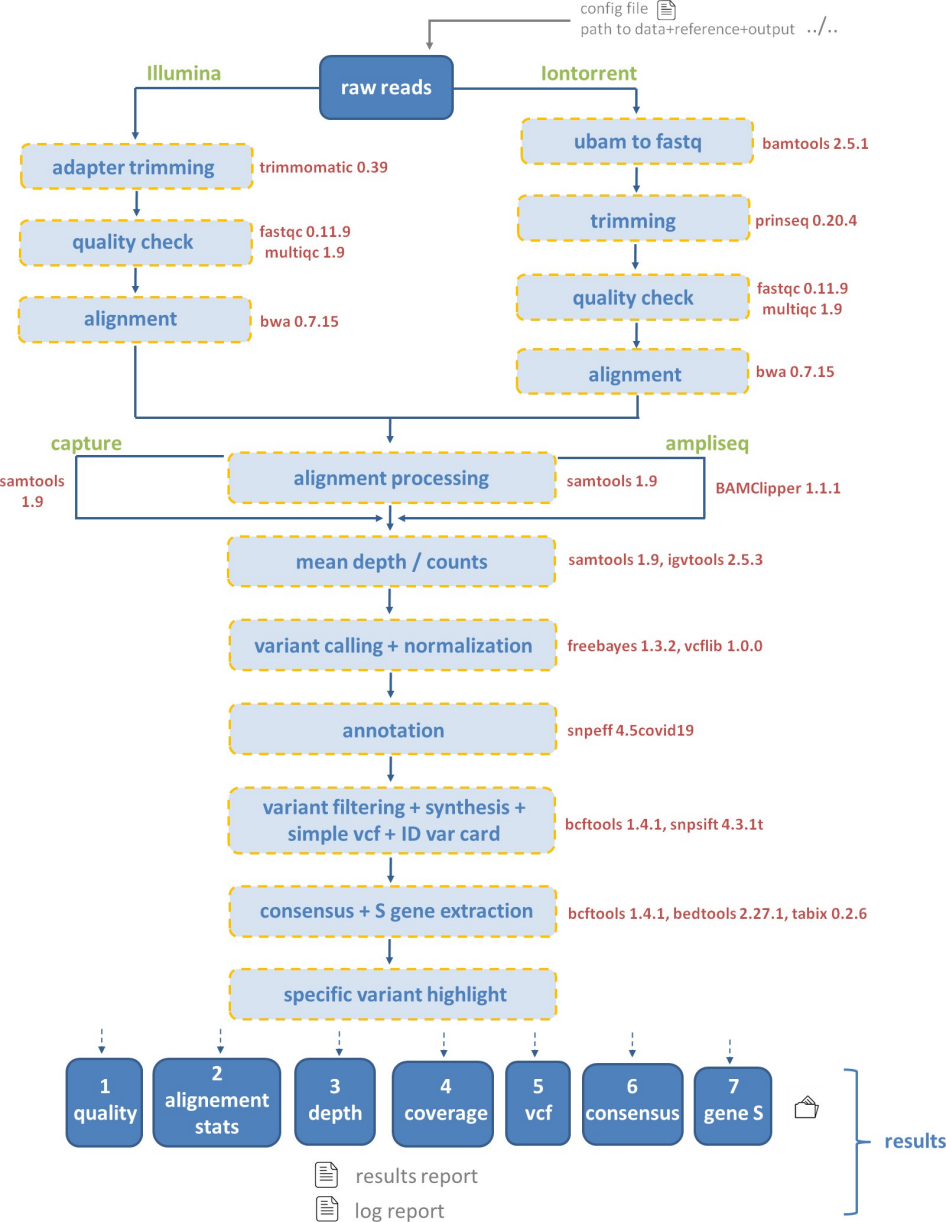
Overview of ASPICov steps and software implemented. In red: Software used, green: Option used.

The succession of genomic tools used (Fig 1) combined to an optimized computing configuration is a key for the robustness of the pipeline.

To facilitate the use of ASPICov and make it highly reproducible, all tools are automatically installed via pre-built singularity images available from the National Oceanographic Data Center operated by Ifremer in France; member of the Research Data Alliance (https://rd-alliance.org) (ftp://ftp.ifremer.fr/ifremer/dataref/bioinfo/sebimer/tools/AspiCov/). These images are built from recipes available as part of the ASPICov source code (https://gitlab.com/vtilloy/aspicov/-/tree/master/containers).

### Input options

ASPICov is designed to be used on Linux distribution and launched with a single command within a cluster job scheduler or locally. Project name, technology, method, path to data, references, Trimmomatic adapter and bedpe files information will be completed by users in a custom configuration file (supported by profile), according to standard Nextflow principles. To use this workflow on a computing cluster, it is necessary to provide an institute configuration file (using -c <institute_config_file>) in order to enable Singularity and to setup the appropriate execution settings for the environment.

### Output files

ASPICov generates different results organized in seven folders (Fig 1). Figures, filtered VCF, specific variant highlight and consensus files are particularly helpful for biological interpretation.

### Availability

ASPICov is a free and open-source pipeline available and updated on a public Gitlab repository (https://gitlab.com/vtilloy/aspicov). It is provided with a quick start guide, a complete documentation describing all options available to fine tune data processing.

### Dataset used to design and to validate the pipeline

Wuhan strain (NC_045512 [5] was used as whole genome reference during pipeline validation. ASPICov has been optimized using a dataset from a single sample (Basa strain isolated from a patient with mild Covid disease at Limoges hospital) taken at different culture stages (P3, P4 and P7 passages), serially diluted (10^−1^, 10^−2^, 10^−3^, 10^−4^, 10^−5^, 10^−6^ and 10^−7^) and processed using Thermofisher and/or Swift Ampliseq protocols, Illumina (S1 Table). We have thus determined a threshold corresponding to background noise: nucleotidic variants were considered as low quality if Phred score is below 200 or depth below 100 or allelic frequency below 0.02. Mutation(s) not retained by filters are still available in VCF files tagged ‘filter’ whereas selected mutations are tagged ‘filter-pass’.

## Results

### ASPICov validation

We have screened ENA and SRA public databases to get a dataset of SARS-Cov2 reads coming from different labs using different strategies and sequencing technologies. Our aim was to validate ASPICov from a wide range of data. All VOC and VOI were found using ASPICov workflow, demonstrating its efficiency and accuracy (S2 Table).

### ASPICov potential applications

From filter optimization we were able to finely observe and intersect changes for a single sample at different culture passages.

We were also able to evaluate repeatability of sequencing methods by sequencing the same library on two runs with the same sequencing technology (Ion Torrent) and also by comparing two strategies (ThermoFisher and Swift amplicons designs).

## Conclusions

ASPICov pipeline is dedicated to detect and identify finely SARS-Cov2 mutations from a broad range of parameters (various samples, different sequencing approaches) with concrete applications in diagnostic and wastewater domains. In order to ensure FAIR data analysis, the workflow is built as a Nexflow pipeline, follows nf-core guidelines and use Singularity containers to wrap tool environments. Its efficiency and accuracy have been demonstrated.

Due to detection of VOI/VOC and IonTorrent technology analysis, ASPICov is complementary to other pipeline such as viralrecon [6] and Farkas pipeline [7]. Conception is different allowing to have an alternative and also a contribution to the diversity of tools for whole genome covid analysis.

ASPICov is regularly updated on Gitlab for special variants according to WHO publications.

Several new features are currently under development, such as a global HTML report, phylogenetic analysis, integration of ONT and MGI sequencing technologies, highlight of genotype percentage, PANGO lineage determination and Nextclade/Gisaid data comparison.

## Supporting information

S1 Table Description of samples used in the study. (DOCX)

S2 Table SARS-Cov2 variants of concern (VOC) and interest (VOI). (DOCX)
