# Supplementary material for: ASPICov: An automated pipeline for identification of SARS-Cov2 nucleotidic variants
Source: PLoS One. 2022 Jan 26;17(1):e0262953. doi: 10.1371/journal.pone.0262953 (PMC8791494; doi:10.1371/journal.pone.0262953)
Supplement: S1 Table — (DOCX) [file pone.0262953.s001.docx]

**S1 Table. Description of samples used in the study**

| **SRA number** | **strain** | **dilution** | **culture passage** | **copies/mL covid** | **sequencing technology** | **library strategy** | **sequencing run** | **reference** |
| --- | --- | --- | --- | --- | --- | --- | --- | --- |
| NC_045512.2 | Wuhan | - | - | - | Illumina | - | - | [5] |
| SAMN20059061 | basa | 10-1 | P3 | 6,22E+09 | Illumina | amplicon Swift | MiSeq-run-1 | this study |
| SAMN20059062 | basa | 10-2 | P4 | 9,43E+09 | Illumina | amplicon Swift | MiSeq-run-1 | this study |
| SAMN20059063 | basa | 10-3 | P4 | 9,43E+08 | Illumina | amplicon Swift | MiSeq-run-1 | this study |
| SAMN20059064 | basa | 10-4 | P4 | 9,43E+07 | Illumina | amplicon Swift | MiSeq-run-1 | this study |
| SAMN20059065 | basa | 10-5 | P4 | 9,43E+06 | Illumina | amplicon Swift | MiSeq-run-1 | this study |
| SAMN20059066 | basa | 10-6 | P4 | 9,43E+05 | Illumina | amplicon Swift | MiSeq-run-1 | this study |
| SAMN20059067 | basa | 10-7 | P4 | 9,43E+04 | Illumina | amplicon Swift | MiSeq-run-1 | this study |
| SAMN20059068 | basa | 10-1 | P3 | 6,22E+09 | Ion Torrent | amplicon Ion Torrent | S5-run-1 | this study |
| SAMN20059069 | basa | 10-2 | P4 | 9,43E+09 | Ion Torrent | amplicon Ion Torrent | S5-run-1 | this study |
| SAMN20059070 | basa | 10-3 | P4 | 9,43E+08 | Ion Torrent | amplicon Ion Torrent | S5-run-1 | this study |
| SAMN20059071 | basa | 10-4 | P4 | 9,43E+07 | Ion Torrent | amplicon Ion Torrent | S5-run-1 | this study |
| SAMN20059072 | basa | 10-5 | P4 | 9,43E+06 | Ion Torrent | amplicon Ion Torrent | S5-run-1 | this study |
| SAMN20059073 | basa | 10-6 | P4 | 9,43E+05 | Ion Torrent | amplicon Ion Torrent | S5-run-1 | this study |
| SAMN20059074 | basa | 10-7 | P4 | 9,43E+04 | Ion Torrent | amplicon Ion Torrent | S5-run-1 | this study |
| SAMN20059075 | basa | none | P7 | 6,57E+09 | Ion Torrent | amplicon Ion Torrent | S5-run-2 | this study |
| SAMN20059076 | basa | 10-1 | P3 | 6,22E+09 | Ion Torrent | amplicon Ion Torrent | S5-run-2 | this study |
| SAMN20059077 | basa | none | P7 | 6,57E+09 | Illumina | amplicon Swift | MiSeq-run-2 | this study |
