# Supplementary material for: ASPICov: An automated pipeline for identification of SARS-Cov2 nucleotidic variants
Source: PLoS One. 2022 Jan 26;17(1):e0262953. doi: 10.1371/journal.pone.0262953 (PMC8791494; doi:10.1371/journal.pone.0262953)
Supplement: S2 Table — (DOCX) [file pone.0262953.s002.docx]

**S2 Table. SARS-Cov2 variants of concern (VOC) and interest (VOI).**

|  | **WHO label** | **Pango lineage** | **GISAID clade/**  **lineage** | **Nextstrain**  **clade** | **Earliest documented**  **samples** | **Date of designation** | | **Notable mutation** | **Bio-sample** | **Information** | **Mutations found with ASPICov** |
| --- | --- | --- | --- | --- | --- | --- | --- | --- | --- | --- | --- |
| Variants of concern | Alpha | B.1.1.7 | GRY (formerly GR/501Y.V1) | 20I/S:501Y.V1 | UK, Sep-2020 | 18-Dec-2020 | 69–70del, N501Y, P681H | | SAMN18219363 | amplicon COVIDSeq / Illumina NovaSeq | 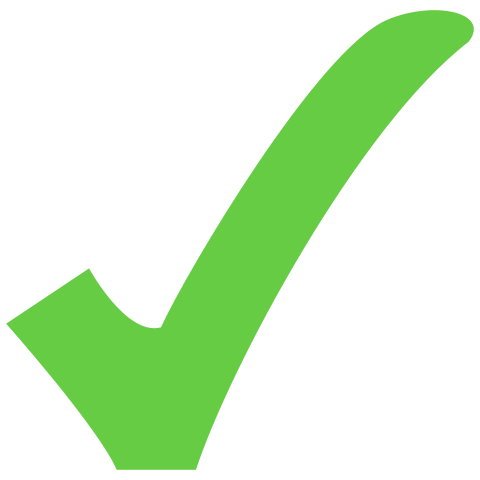 |
|  | Beta | B.1.351 | GH/501Y.V2 | 20H/S:501Y.V2 | South Africa, May-2020 | 18-Dec-2020 | K417N, E484K, N501Y | | SAMN17767551 | amplicon Artic / Illumina MiSeq | 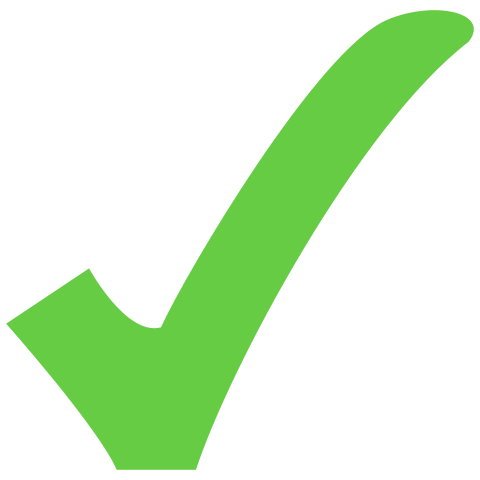 |
|  | Gamma | P.1 | GR/501Y.V3 | 20J/S:501Y.V3 | Brazil, Nov-2020 | 11-Jan-2021 | K417T, E484K, N501Y | | SAMN18527803 | ? / Illumina MiSeq | 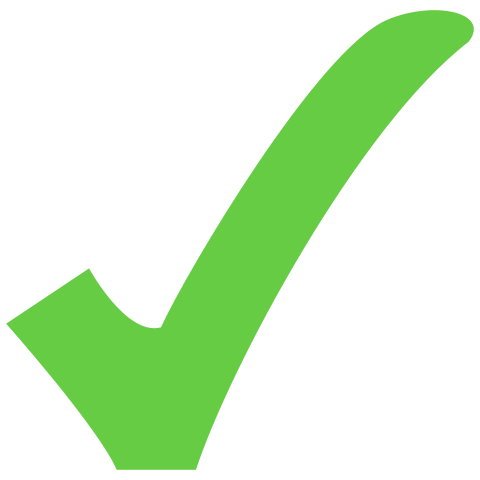 |
|  | Delta | B.1.617.2 | G/452R.V3 | 21A/S:478K | India, Oct-2020 | VOI: 4-Apr-2021  VOC: 11-May-2021 | L452R, T478K, P681R | | SAMN19157798 | artic Amplicon / Illumina | 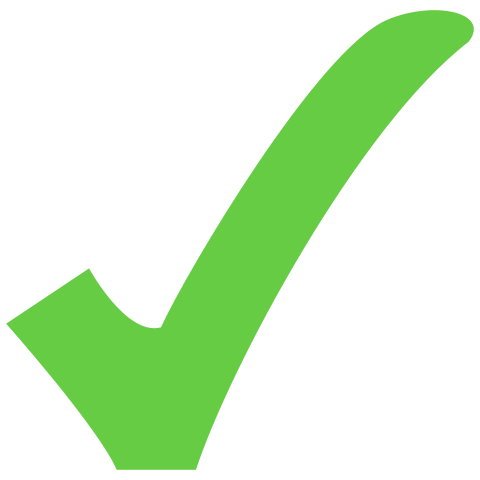 |
| Variants of interest | Epsilon | B.1.427 / B.1.429 | GH/452R.V1 | 20C/S.452R | USA, Mar-2020 | 5-Mar-2021 | L452R | | SAMN19223936 | amplicon Artic / Illumina Nextera | 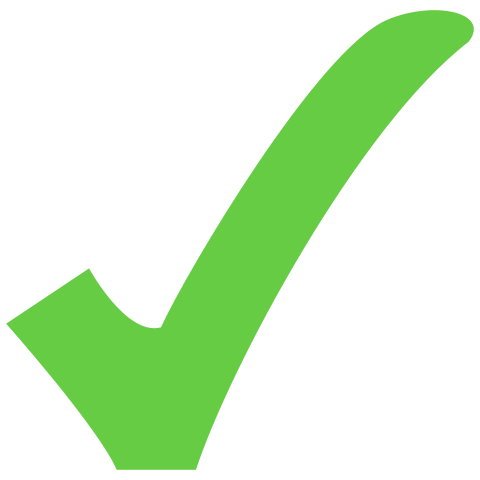 |
|  | Zeta | P.2 | GR | 20B/S.484K | Brazil, Apr-2020 | 17-Mar-2021 | E484K | | SAMN18606318 | amplicon Artic / Illumina NovaSeq | 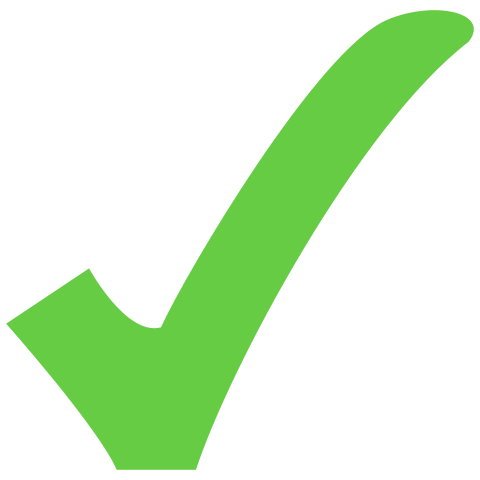 |
|  | Eta | B.1.525 | G/484K.V3 | 20A/S484K | Multiple countries, Dec-2020 | 17-Mar-2021 | E484K, F888L | | SAMEA7763427 | amplicon / Illumina NovaSeq | 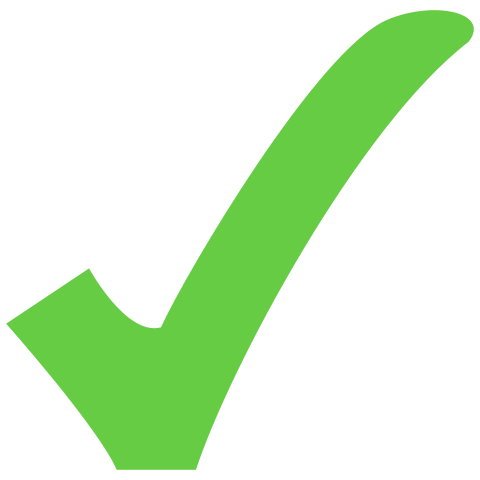 |
|  | Theta | P.3 | GR | 20B/S:265C | Philippines, Jan-2021 | 24-Mar-2021 | E484K, N501Y, D614G, P681H, E1092K, H1101Y, V1176F | | SAMEA9094797 | amplicon / Illumina NovaSeq | 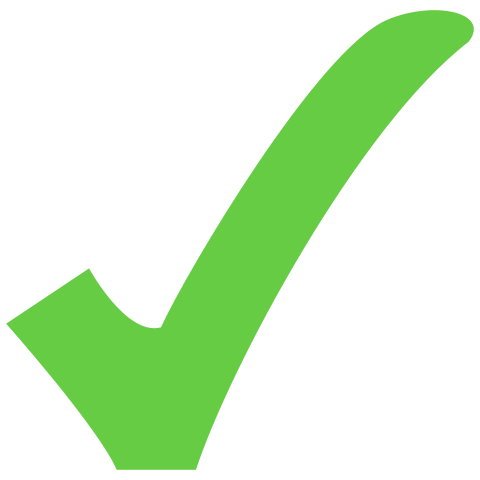 |
|  | Iota | B.1.526 | GH | 20C/S:484K | USA, Nov-2020 | 24-Mar-2021 | E484K | | SAMN19009806 | amplicon Artic / Illumina NovaSeq | 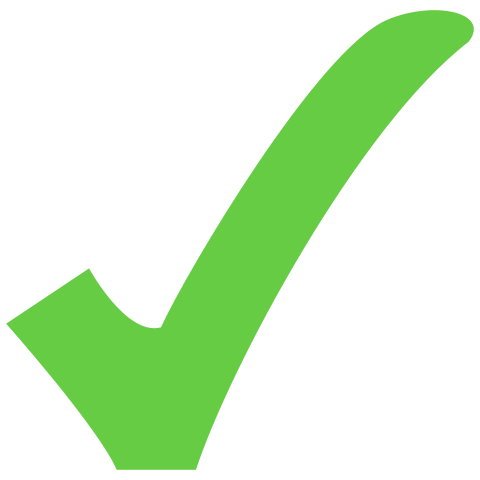 |
|  | Kappa | B.1.617.1 | G/452R.V3 | 21A/S:154K | India, Oct-2020 | 4-Apr-2021 | L452R, E484Q, P681R | | SAMN19375761 | amplicon Thermo / S5 Iontorrent | 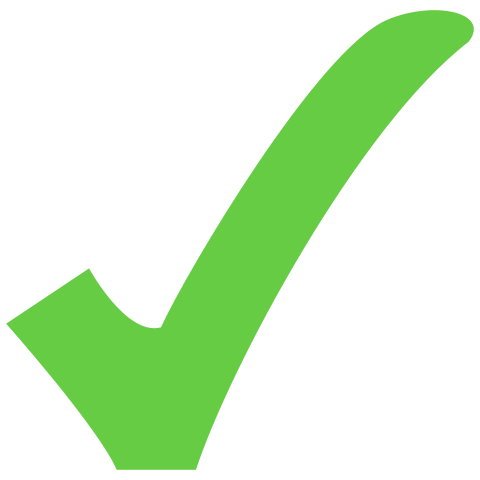 |
|  | Lambda | C.37 | GR/452Q.V1 | 20D | Peru, Aug-2020 | 14-June-2021 | L452Q | | SAMN19224075 | amplicon Artic / Illumina Nextera | 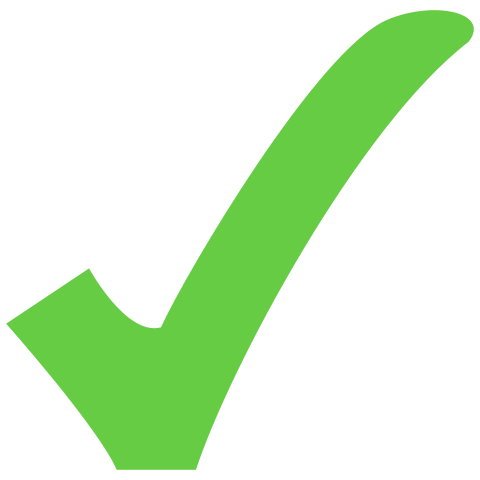 |
|  | Mu | B.1.621 | GH | 21H | Colombia, Jan-2021 | 30-Aug-2021 | T95I, Y144S, Y145N, R346K, E484K, N501Y, D614G, P681H, D950N | | SAMEA9083610 | Amplicon / Illumina MiSeq | 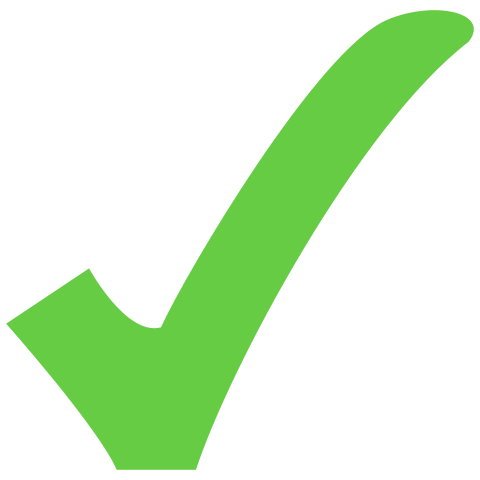   \|  \| \| --- \| |
| Other variant |  | B.1.1.207 |  |  | Nigeria, Aug-2020 | Aug 2020 | P681H | | SAMN17717137 | ? / Illumina TruSeq | 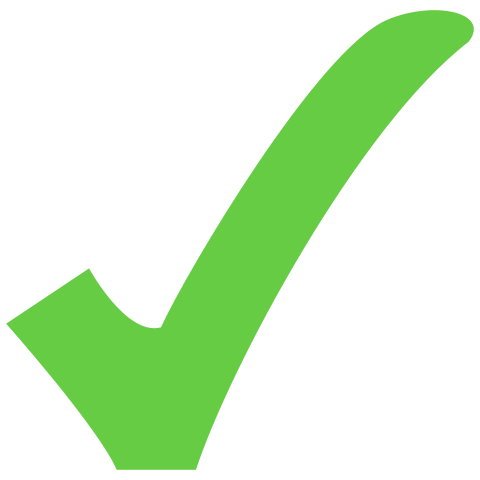 |

Samples from public databases have been tested using ASPICov workflow to see if the the pipeline can detect the VOI or VOC from various technologies. Updated on 17 september 2021. Sources : https://www.who.int/en/activities/tracking-SARS-CoV-2-variants/ and https://en.wikipedia.org/wiki/Variants_of_SARS-CoV-2
